# HetMM: A Michaelis-Menten model for non-homogeneous enzyme mixtures

**DOI:** 10.1016/j.isci.2024.108977

**Published:** 2024-01-19

**Authors:** Jordan Douglas, Charles W. Carter, Peter R. Wills

**Affiliations:** 1 Department of Physics, The University of Auckland, Auckland 1010, New Zealand; 2 Centre for Computational Evolution, The University of Auckland, Auckland 1010, New Zealand; 3 Department of Biochemistry and Biophysics, University of North Carolina at Chapel Hill, NC 27599, USA

**Keywords:** Computational chemistry, Biochemistry, Biomolecules, Protein

## Abstract

The Michaelis-Menten model requires its reaction velocities to come from a preparation of homogeneous enzymes, with identical or near-identical catalytic activities. However, this condition is not always met. We introduce a kinetic model that relaxes this requirement, by assuming there are an unknown number of enzyme species drawn from a probability distribution whose standard deviation is estimated. Through simulation studies, we demonstrate the method accurately discriminates between homogeneous and heterogeneous data, even with moderate levels of experimental error. We applied this model to three homogeneous and three heterogeneous biological systems, showing that the standard and heterogeneous models outperform respectively. Lastly, we show that heterogeneity is not readily distinguished from negatively cooperative binding under the Hill model. These two distinct attributes—inequality in catalytic ability and interference between binding sites—yield similar Michaelis-Menten curves that are not readily resolved without further experimentation. Our user-friendly software package allows homogeneity testing and parameter estimation.

## Highlights

• The HetMM model describes the kinetics of a mixture of enzymes with varying K_m_
• Accurately discriminates between homogeneous and nonhomogeneous systems
• Tolerant to moderate levels of experimental error
• Negative cooperative binding under the Hill model gives the false appearance of a mixture

## Graphical abstract

**Figure:**
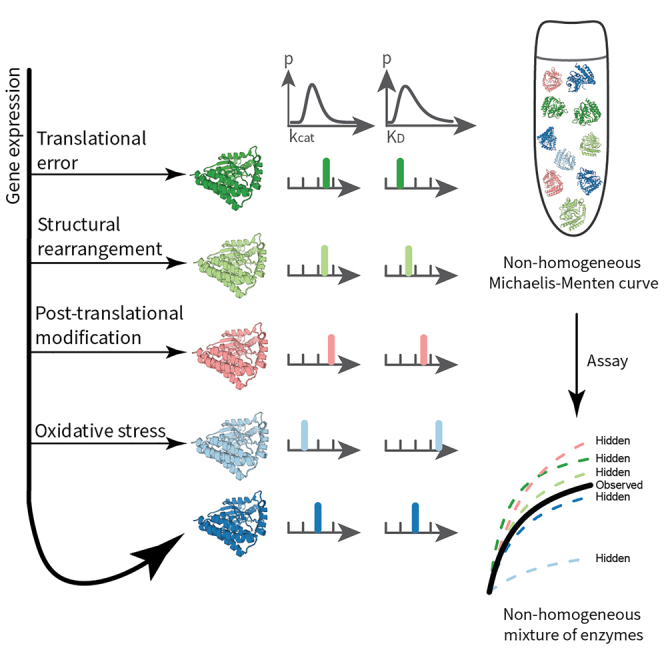

## Introduction

One hundred and ten years after its initial publication,^1^ the equation devised by Leonor Michaelis and Maud Menten is still routinely applied to predicting and explaining biochemical systems, to the point where it is almost synonymous with enzyme kinetics. And like all models, the Michaelis-Menten (MM) model makes several assumptions about the conditions that generated the observed data (see review by Srinivasan 2022^2^). It is assumed that the enzyme concentration is many orders of magnitude less than its substrate,^3^ and the latter is assumed to remain stationary for the observed duration of the reaction,^4^ as does the concentration of the enzyme-substrate complex in the quasi-steady state that characterizes the model.^5^

**Figure 1:**
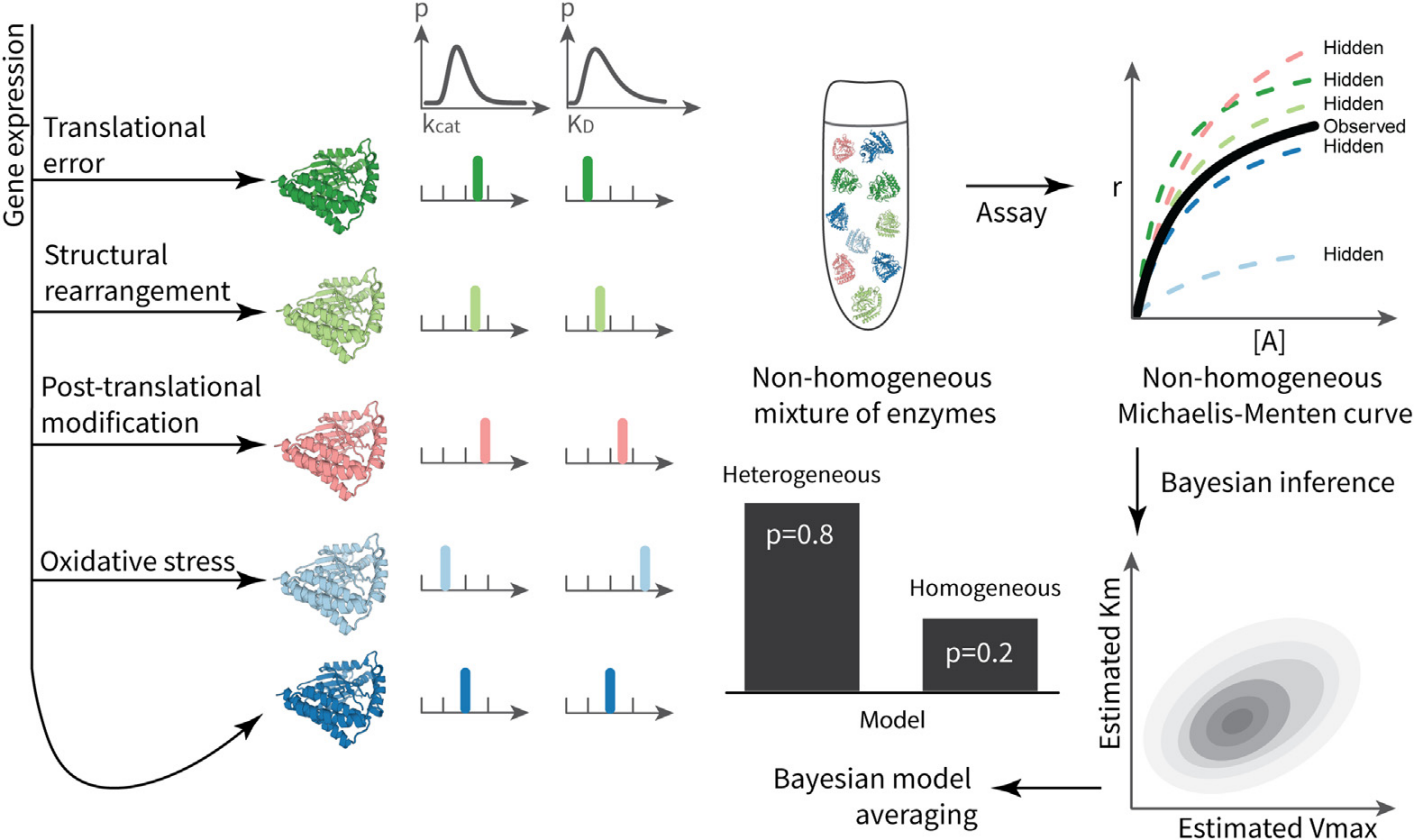
Capturing enzyme heterogeneity Left: several mechanisms that can lead to enzyme heterogeneity. The properties of each enzyme, $k_{cat}$ and $K_{D}$, are variable and can be described by some unknown probability distribution (with probability density *p*), shown at the top. Right: flowchart for testing for homogeneity. The end result is a posterior probability of either model (homogeneous and heterogeneous) being the correct model for the dataset, as well as parameter estimates.

Another fundamental assumption, this one so intuitively obvious that it can be easily overlooked, is that the catalysts (and reactants) are homogeneous. That is, the solution is composed of an ensemble of catalysts, each of which displays identical, or near-identical, catalytic activity. However, this condition is often violated in biological systems, even in purified enzyme preparations.^6^^,^^7^ There may exist multiple enzyme species in the preparation, each with distinct catalytic rates $k_{\text{cat}}$ and dissociation constants $K_{D}$. In such a scenario, it would be preferable to characterize the kinetics of the catalyst, not as a single entity, but as a *mixture* of catalytic agents, whose catalytic properties are described by some probability distribution (Figure 1).

Indeed, there are numerous cases of nonhomogeneous biocatalytic systems, both *in vitro* and *in vivo*. Many genomes express isozymes; enzymes with distinct sequences, but common ancestry and common catalytic activity, such as the multiple aminoacyl-tRNA synthetase gene duplicates that coexist in bacterial cells.^8^ In some cases, purified enzyme preparations contain multiple isozymes, such as cytochrome oxidase.^7^^,^^9^ But even a single gene can give rise to a heterogeneous mixture of protein products, through transcriptional or translational errors, alternative initiation or splicing, or post-translational modification, among other processes. Otherwise-rare occurrences can be amplified by the environment, for example thermophilic Archaea can adapt to lower temperatures by increasing the rate of methionine-for-leucine mistranslations.^10^ Primordial proteins in particular were most likely produced by a low-fidelity and ambiguous genetic code, and hence would have been highly variable.^11^^,^^12^ And even proteins with identical sequences, devoid of post-translational modifications, can still fold into a population of distinct structures^13^^,^^14^ with a population of catalytic abilities,^15^ or lack thereof - for instance ion channels^16^ and molten globular enzymes.^17^ Any of these sources of variability could be further compounded by various degradation effects that may emerge from natural biological processes or from an ill-prepared *in vitro* sample. Catalase, for instance, is known as a “suicide” catalyst because it is degraded by its own substrate.^18^ That substrate is hydrogen peroxide, a reactive agent that oxidizes proteins; cysteine side chains in particular.^19^^,^^20^ Further examples of heterogeneity are illustrated by Brown et al. 2014.^7^

On a coarser scale, heterogeneity may be an intrinsic property of the system that cannot be reduced to some mere idiosyncrasy of a particular gene or protein.^21^ This may be the case when studying cells, organelles, or multicellular organisms. When describing such a system with the MM model, the ‘enzyme’ would be a series of complex *in vivo* processes, and the ‘enzyme preparation’ may be living tissue. For instance, Devaux et al. 2023^22^ applied the MM model to quantify oxygen consumption in fish brains.

Generally, heterogeneity is something to avoid in experimental design, but in some cases it may be desirable or even unavoidable. Suppose that not just one, but rather a large sample of enzymes, were to be co-expressed, co-purified, and co-assayed as part of a combinatorial expression library. The assay would measure the kinetic properties of the *population* of forms rather than just one enzyme species. For instance, experimental investigations into ancestral models typically operate on one enzyme sequence at a time,^23^^,^^24^^,^^25^^,^^26^ but this approach could benefit from co-assaying a sample of reconstructions (such as a phylogenetic posterior distribution) to improve the robustness and statistical rigor of the results in a cost-affordable manner.

Some enzymes, such as aspartate kinase^27^ and UDP-GlcNAc 2-epimerase,^28^ display negative cooperativity between binding sites, where binding at one site interferes with the binding affinities at other sites. These interactions are typically modeled using the Hill equation, a generalization of the MM model.^29^^,^^30^^,^^31^ However, as discussed by Abeliovich 2005, negative cooperativity is not readily distinguished from a mixture of independent but variable binding sites.^32^ They propose the 1/N rule, where a Hill coefficient less than the reciprocal of the number of binding sites likely arose from heterogeneity, rather than negative cooperativity.

Clearly, there are many instances where the standard homogeneous model is unjustifiable, and many more instances where one may wish to test the validity of this assumption. In this article we describe an extension to the Michaelis-Menten model, in which it is assumed that there are an unknown number of catalytic species whose properties are independently drawn from an unknown probability distribution, and thus the enzyme preparation is heterogeneous. We provide a Bayesian model averaging framework, to test whether the standard assumption of homogeneity is adequate, or whether the heterogeneous model is preferred. Lastly, we explore the interactions between the heterogeneous and Hill models, and discuss the limitations of either approach. These methods are implemented in the user-friendly, open-source heterogeneous Michaelis-Menten (HetMM) package, which employs the BEAST 2 Bayesian inference engine.^33^ HetMM is available online at https://github.com/jordandouglas/HetMM.

## Results

### Homogeneous and heterogeneous models

Consider the following reaction scheme between substrate *A*, enzyme *E*, and product *P*.

(Equation 1) $$E+A\overset{k_{1}}{\underset{k_{-1}}{⇌}}EA\overset{k_{cat}}{\to }E+P$$

Under the standard (homogeneous) MM model, the expected reaction velocity $\hat{r}$ is equal to

(Equation 2) $$\hat{r}\left(a\right)=\frac{dP}{dt}=\frac{va}{k+a}\;\text{at}\;\text{time}\;t=0$$

for substrate concentration *a*, where *v* is shorthand for $V_{\max}$ (the maximum reaction rate) and *k* for the Michaelis constant $K_{m}$ (the substrate concentration where $\hat{r}$ is half of $V_{\max}$). Under the steady-state approximation, *k* is defined as:

(Equation 3) $$k=\frac{k_{-1}+k_{cat}}{k_{1}}\approx \frac{k_{-1}}{k_{1}}=K_{D}\;\text{when}\;k_{cat}\;\text{is}\;\text{small.}$$

The interpretation of *k* may change if any of the standard Michaelis-Menten assumptions^2^ are not satisfied. Here, we will denote the natural logarithms of *v* and *k* by their uppercase symbols V=logv and K=logk. The transformed parameters *V* and *K* are not necessarily positive. Now, let us assume that *V* and *K* are independently sampled from normal distributions, or equivalently, *v* and *k* are independently sampled from log-normal distributions:

(Equation 4) $$V∼\text{Normal}\left(\mu_{V},\sigma_{V}\right)\equiv v∼\mathrm{Log}–\text{normal}\left(\mu_{V},\sigma_{V}\right)$$

(Equation 5) $$K∼\text{Normal}\left(\mu_{K},\sigma_{K}\right)\equiv k∼\mathrm{Log}–\text{normal}\left(\mu_{K},\sigma_{K}\right)$$

Here, μ∈R and σ>0 are the respective means and standard deviations of *V* and *K*. These two normal distributions have probability densities $f_{V}$ and $f_{K}$. Now let us marginalise across all values of *K* and *V* according to this distribution:

(Equation 6) $$\hat{r}\left(a\right)=\int_{V}\int_{K}\frac{e^{V}a}{e^{K}+a}\;f_{V}\left(V\right)f_{K}\left(K\right)\;dVdK$$

(Equation 7) $$=a\int_{V}\left(e^{V}f_{V}\left(V\right)\;dV\right)\;\int_{K}\left(\frac{1}{e^{K}+a}f_{K}\left(K\right)\;dK\right)$$

(Equation 8) $$=a\;g\left(V\right)\int_{K}\left(\frac{1}{e^{K}+a}f_{K}\left(K\right)\;dK\right)$$

for some function g(V). As this function is independent of *a*, it is clear that any degree of variation in *V* cannot be resolved here without further information, such as the relative abundances of the species. Going forward, we will define $v_{0}$ as the mean $V_{\max}$ across the species, weighted according to their unknown abundances. However, this simplification is not possible in the case of *K*.^7^

(Equation 9) $$\hat{r}\left(a\right)=v_{0}\;a\int_{K}\frac{1}{e^{K}+a}\;f_{K}\left(K\right)\;dK=v_{0}\;a\int_{K}\frac{1}{e^{K}+a}\;\frac{1}{\sigma_{K}\sqrt{2\pi }}\;\exp\;\left(-\frac{1}{2}{\left(\frac{K-\mu_{K}}{\sigma_{K}}\right)}^{2}\right)\;dK$$

**Figure 2:**
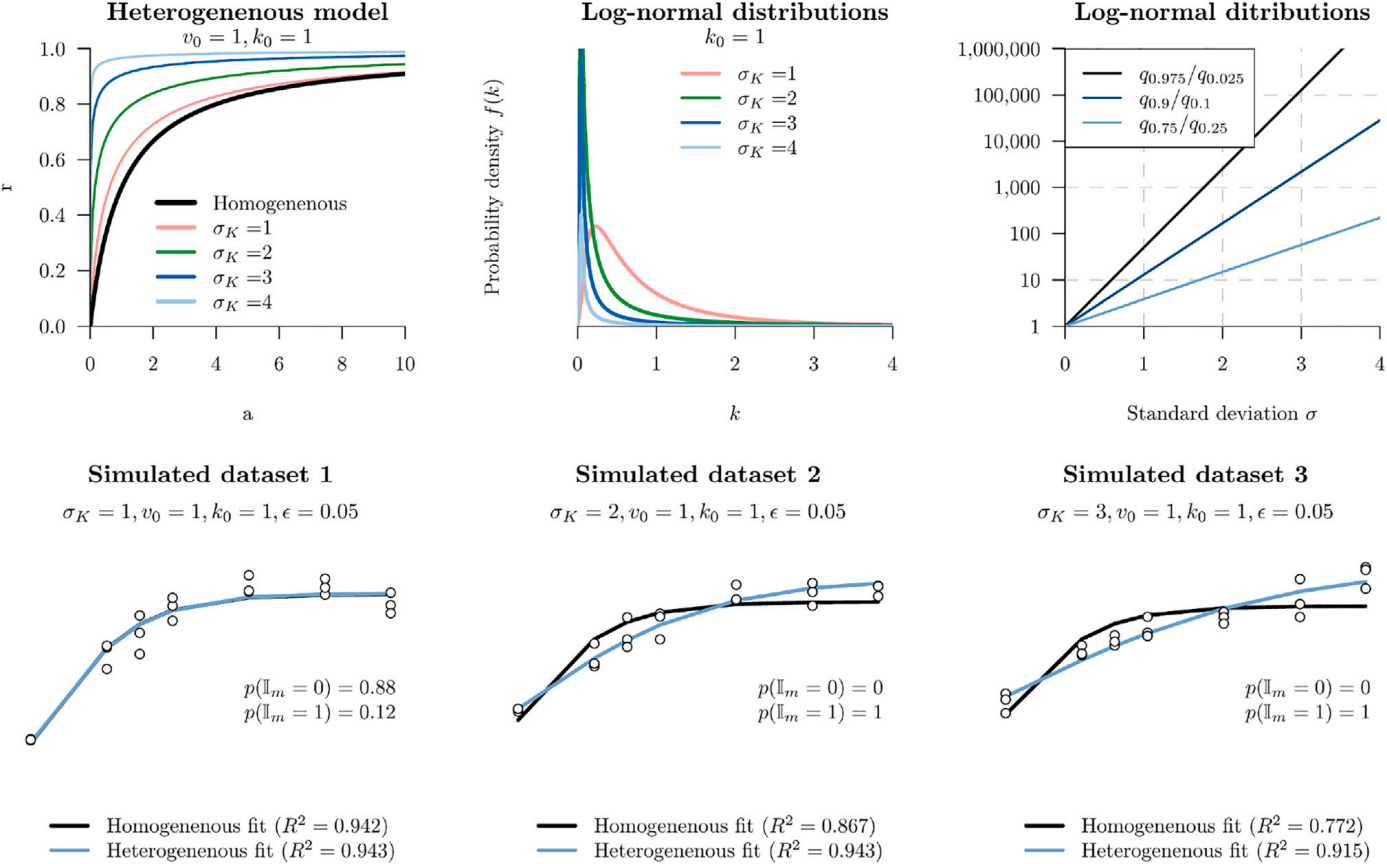
Characterizing the effect of $\sigma_{K}$ on the heterogeneous model Top row: varying $\sigma_{K}$ while keeping all other parameters constant changes the Michaelis-Menten curve. This parameter is the standard deviation of a log-normal distribution. The relative difference between upper and lower quantiles *q*, for varying standard deviations, are shown in the top right figure. Bottom row: three datasets were simulated under the heterogeneous model, with varying $\sigma_{K}$. The x axes are concentrations *a* (log-scale) and the y axes are reaction velocities *r*, which were simulated with random error ϵ. Bayesian inference was run on these datasets, with both models (homogeneous and heterogeneous). In the second two cases ($\sigma_{K}=2,3$), the heterogeneous model is best, because $p\left(I_{m}=1\right)$ is large. However, when $\sigma_{K}=1$, the homogeneous and heterogeneous model could not be discriminated between, and therefore the model averaging favored the simpler model $I_{m}=0$.

A numerical approximation of this integral is used. This new parameter, $\sigma_{K}$, describes the degree of variability in *k*. As $\sigma_{K}$ approaches zero, the homogeneous and heterogeneous models become indistinguishable, and as $\sigma_{K}$ grows beyond 1–2, the Michaelis constants *k* span several orders of magnitude (right panel of Figure 2). As confirmed in Figure 2, the Michaelis-Menten curve is sensitive to changes in $\sigma_{K}$ when $\sigma_{K}>1$. Whereas, when $\sigma_{K}>1$, the assumption of homogeneity is often adequate, even if untrue. Overall, this model of heterogeneity assumes there are an unknown number of enzymes whose Michaelis constants fall onto a continuous spectrum of binding affinities and catalytic rates. In practice, the various *k* values of the mixture need not be log-normally distributed, however this flexible distribution is significantly more relaxed than the assumption of all catalytic species having identical *k*. This continuous model differs from the heterogeneity model by Brown et al. 2014,^7^ which assumes a small number of individually homogeneous species (e.g., two species) that are weighted according to their relative abundances.

### How heterogeneous is heterogeneous?

Before the distinction between homogeneity and heterogeneity makes any sense, we must first quantify how disparate the catalytic species should be so that their population is observably nonhomogeneous. Suppose, for instance, that an ensemble of enzymes were composed of several species, whose $K_{m}$ ranged from 1 to 1.1 units (if the species were to be characterized in isolation). In this case, the system would be effectively homogeneous. As demonstrated by Brown et al. 2014, the assumption of homogeneity is likely sufficient for the case of two species, provided that the ratio between their $K_{m}$ is no more than 20.^7^ Moreover, as shown in Figure 2 (bottom right), the standard deviation $\sigma_{K}$ should be >1 in order for the relative difference between the upper and lower extremes of *k* (among the population of enzymes) to be multiple orders of magnitude apart, and for the two models to have distinguishable properties. This logic forms the basis of our prior distribution for $\sigma_{K}$, which is shown in Table 1 along with the other priors.

**Table 1:** Prior distributions used in this study

Parameter	Prior	Notes
*General case*		
$v_{0}$	Log-normal(μ=1,σ=2)	Uninformative prior
$k_{0}$	Log-normal(μ=1,σ=2)	Uninformative prior
$\sigma_{K}$	Log-normal(μ=1,σ=0.5)	Ensures the heterogeneous model is “heterogeneous”
ϵ	Log-normal(μ=−1.5,σ=1)	A reasonable level of experimental error
$I_{m}$	Uniform({0,1})	Both models are equal *a priori*
*Hill model*		
$h_{0}$	Beta(α=2,β=4)	Shifts *h* away from 1
$I_{h}$	Uniform({−1,0,0,1})	Hill and not-Hill are equal *a priori*
*Simulation studies*		
$v_{0}$	Log-normal(μ=1,σ=0.5)	
$v_{0}$	Log-normal(μ=1,σ=0.5)	
*Hb dataset*		
$v_{0}$	100	Fixed at 100% saturation
*MnP dataset*		
$I_{h}$	0	Hill model disabled (only one active site)
*CFTR dataset*		
$h_{0}$	Beta(α=30,β=10)	Low probability of h<0.5 or h>2 (two active sites)

Note that a Log-normal distribution’s μ and σ are the mean and standard deviation in log-space. The mean in regular space is $\log\;\mu -\frac{\sigma^{2}}{2}$. In a real-world example, the priors for $v_{0}$ and $k_{0}$ should be adjusted to reflect the units of measurement and other *a priori* information about the system being studied.

### Bayesian inference and model averaging

Parameter estimation is performed using Bayesian inference, allowing for robust estimation of parameters and their credible intervals, as well as the model indicator $I_{m}\in \left\{0,1\right\}$, which governs the use of the homogeneous or heterogeneous MM model. This approach is known as Bayesian model averaging.^34^^,^^35^ It enables robust and probabilistic estimation of the model as if it were a parameter. Much like the Akaike and Bayesian information criteria, the method penalizes overparameterized models. Let *D* be the data measured from a kinetic assay, consisting of a vector of substrate concentrations $a=\left(a_{1},a_{2},\ldots ,a_{n}\right)$ and empirical reaction rates $r=\left(r_{1},r_{2},\ldots ,r_{n}\right)$. Given model parameters θ, the likelihood is calculated by comparing observations *r* with their expected values $\hat{r}$, conditional on substrate concentration *a*, using the equation

(Equation 10) $$p\left(D|\theta \right)=\prod_{i=1}^{n}f_{\epsilon }\left(\log\hat{r}\left(a_{i}\right)-\log\;r_{i}\right)$$

where the experimental errors between expected $\hat{r}\left(a\right)$ and observed *r* values are assumed to follow a normal distribution, in log-space. This normal distribution, with probability density $f_{\epsilon }$, has mean 0 and standard deviation ϵ. Modeling experimental error in log-space accounts for the fact that reaction rates are non-negative, while also accommodating for the common scenario where the magnitude of error increases with substrate concentration, i.e., heteroscedasticity. Under this parameterization, ϵ describes relative error and is therefore agnostic about measurement units. An error of ϵ=0.2, for example, has the same meaning regardless of whether velocities are in units of M per second or μM per second. This model consists of five parameters $\theta =\left(v_{0},k_{0},\sigma_{K},\epsilon ,I_{m}\right)$. If $I_{m}=0$, then the enzymes are assumed to be homogeneous, in which case $\hat{r}$ is calculated using Equation 2, where $v=v_{0}$ and $k=k_{0}$. $\sigma_{K}$ is not being used in the model. Whereas, if $I_{m}=1$, then the enzymes are assumed to be heterogeneous, in which case $\hat{r}$ is calculated using Equation 9, where $\mu_{K}=\log\;k_{0}-\frac{1}{2}\sigma_{K}^{2}$, so that $\mu_{K}$ reflects the mean of *k* in real-space, rather than log-space. In this model, all five model parameters in θ are being used. Lastly, the posterior density is

(Equation 11) p(θ|D)∝p(D|θ)×p(θ).

Our prior distributions p(θ) are summarised in Table 1. The posterior distribution is sampled using Markov chain Monte Carlo (MCMC), as implemented in BEAST 2.^33^ Although BEAST 2 was designed for phylogenetic inference, its use of efficient proposal kernels, including Bactrian,^36^ adaptable variance multivariate normal,^37^ and adaptable operator sampler^38^ kernels, makes it an attractive engine for the purposes of this model. MCMC chains are run until the effective sample sizes of all parameters exceed 200, as diagnosed by Tracer.^39^

Lastly, we wish to address a point of potential confusion concerning the varying use of log-normal distributions. We have used log-normal distributions in three different contexts here. First, in Equation 5, we assume that the properties of enzymes in the population are distributed in a log-normal fashion, i.e., log-normal $\left(\mu_{K},\sigma_{K}\right)$. Second, in Equation 10, we assume the experimental error is drawn from log-normal (0,ϵ) - this distribution describes random error in the experimentation process. Lastly, in Table 1, we use the log-normal as prior distributions - these distributions describe information about any *a priori* expectations of a parameter, before performing any analyses. As shown in Figure 2, log-normal distributions are flexible, they have positive domains, and their shapes are determined by only one parameter, σ, making them readily interpretable.

### Hill model extension

We describe a heterogeneous extension to the Hill model. The Hill equation is an empirical model that allows interaction between binding sites or the compartments of a tissue, e.g., muscle.^29^^,^^30^^,^^31^ The nature and degree of this interaction is described by the Hill coefficient h>0:

(Equation 12) $$\hat{r}\left(a\right)=\frac{va^{h}}{k^{h}+a^{h}}$$

When h>1, this indicates positive cooperativity between binding sites (giving a sigmoidal MM curve), and h>1 indicates negative cooperativity (giving an MM-like curve with a sharpened hyperbola). When h=1, there is no interaction, and the Hill model is equivalent to MM. Many have argued that the Hill model is merely a descriptive model, and lacks a strong theoretical basis.^31^ In any case, like the MM model, the Hill model assumes homogeneity. Thus, under the heterogeneous Hill model:

(Equation 13) $$\hat{r}\left(a\right)=\int_{K}\frac{va^{h}}{e^{Kh}+a^{h}}\;f_{K}\left(K\right)\;dK$$

The posterior distribution of this model $\theta_{h}$ is estimated using MCMC from the posterior density (Equation 11). However, there are two additional parameters in $\theta_{h}$ that are absent from the heterogeneous MM model. These two parameters govern whether the Hill coefficient is positive, negative, or neutral. *h* is equal to $h_{0}$ when $I_{h}=-1$, 1 when $I_{h}=0$, and $\frac{1}{h_{0}}$ when $I_{h}=1$, where $h_{0}\in \left(0,1\right)$ is the Hill coefficient in the case of negative cooperativity, and its reciprocal for positive cooperativity. This parameterization ensures symmetry in their prior distributions, and places the probability mass away from h=1 so that the three models are distinct. Moreover, their prior distributions should reflect prior knowledge about the number of binding sites, and place the majority (or all) of their probability densities within the range $\left(\frac{1}{N},1\right)$ and (1,N), respectively, where *N* is the number of binding sites in each enzyme. Inferring the value of the model indicator $I_{h}\in \left\{-1,0,1\right\}$ during MCMC can be used to test whether the Hill model is applicable to a given dataset, and if so, then the direction in which cooperativity acts. This is also known as Bayesian modeling averaging.

### Validation on simulated data: The model is well-calibrated

**Figure 3:**
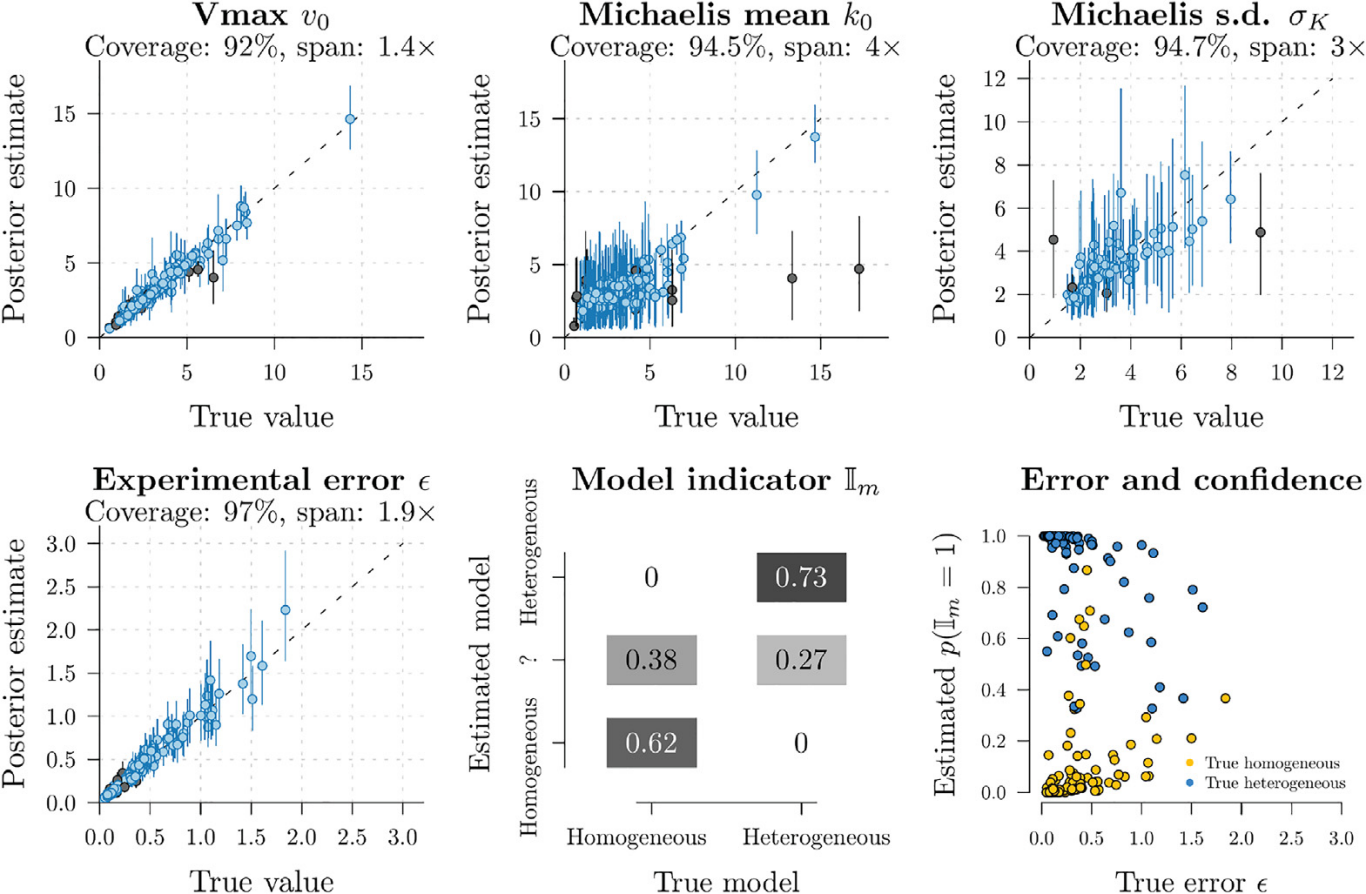
Well-calibrated simulation study (heterogeneous Michaelis-Menten model) Two hundred datasets were simulated, and the parameters used to generate them were recovered with Bayesian MCMC. The coverage is the percentage of simulations where the true parameter lies in its 95% credible interval (blue lines), compared to the times when its estimate is wrong (black lines). The span is the relative difference between the upper and lower 95% interval points, geometrically-averaged across all replicates and rounded to 2 significant figures. These results show that coverage is close to 95% and that the spans are within an order of magnitude, thus providing confidence in the ability of the method to recover parameters. $\sigma_{K}$ is not part of the homogeneous model, and therefore its coverage is only calculated when $p\left(I_{m}=1\right)>0.95$. In the bottom middle panel, the estimated model is indicated if it has more than 0.95 posterior support, while ‘?’ denotes uncertainty (i.e., 0.05<p(I=1)<0.95).

**Figure 4:**
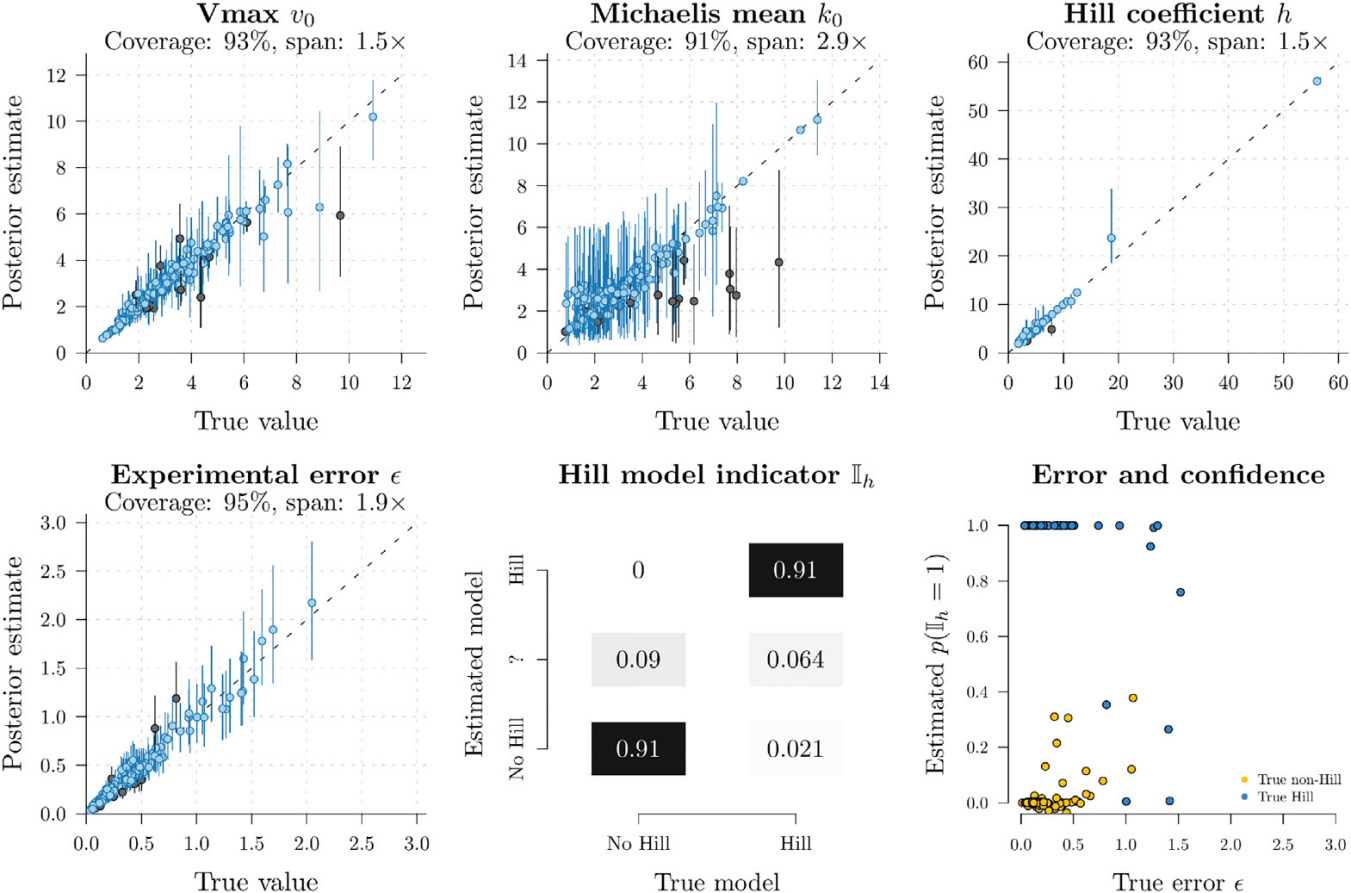
Well-calibrated simulation study (homogeneous Hill model) Each point represents an MCMC chain performed on one of two hundred datasets simulated under known parameters. *h* is part of the Hill model, and therefore its coverage is only calculated when $p\left(I_{h}\neq 0\right)>0.95$. The true estimated model is indicated if it has more than 0.95 posterior support, while ‘?’ denotes uncertainty (i.e., $0.05<p\left(I_{h}\neq 0\right)<0.95$).

In order to test how accurately our method can recover parameters from datasets generated by the same model, we simulated two hundred datasets and recovered the parameters on each of them using Bayesian MCMC. These datasets were simulated using parameters that were randomly sampled from the prior distribution. This experiment was designed to test (1) whether the true parameters can be recovered, and (2) whether the true model (homogeneous or heterogeneous) can be recovered. It would be undesirable if, for instance, the heterogeneous model was disproportionately selected even on homogeneous data. The conditions of these simulations mirrored a typical experimental setup, with three sets of reaction rates independently measured per substrate concentration, across seven different concentrations. These seven concentrations flanked the Michaelis constant *k*, which was sampled from the prior with a mean value of 3 units. These concentrations were 0.5, 2.5, 5, 10, 50, 250, and 1000 units. These results provide confidence that the method is able to recover the true parameters and model. In the case of continuous parameters, the true value was in the 95% credible interval approximately 95% of the time (see Figure 3 coverage). Moreover, the relative differences between lower and upper estimates were sufficiently small that their parameter estimates remained fairly informative (see Figure 3 span). Notably, $V_{\max}$ and ϵ had spans less than two-fold, while the Michaelis mean $k_{0}$ and standard deviation $\sigma_{K}$ had the largest spans (4× and 3×), suggesting they were more challenging to estimate. The spans are likely to widen with less available data. The true model indicator $I_{m}$ was identified, with over 95% support, the majority of the time. In some cases, the model could not be confidently resolved, where $0.05<p\left(I_{m}=1\right)<0.95$. These simulation studies provided no evidence of systematic “overfitting”, where the model with a larger dimensionality (i.e., the heterogeneous model) was favored because it captures random noise. Moreover, discrimination between the two models can be achieved even on datasets with moderate degrees of experimental error, where ϵ≲0.5. The model is therefore quite robust to random error - a standard deviation of ϵ=0.5 means that 95% of the measured reaction velocities at a given concentration can span up to 10-fold, and yet the true model (homogeneous/heterogeneous) can often still be recovered. We performed the same experiment for the homogeneous Hill model and found similar results (Figure 4). Namely, the two models (Hill and not-Hill) could be accurately discriminated between, the parameters could be recovered, and the 95% credible intervals were small enough to be useful. Moreover, detection of Hill binding was even more robust to experimental error than heterogeneity. This stringent validation method (simulation-based calibration^40^) is becoming increasingly performed when testing Bayesian models in biological settings.^41^^,^^42^^,^^43^

**Figure 5:**
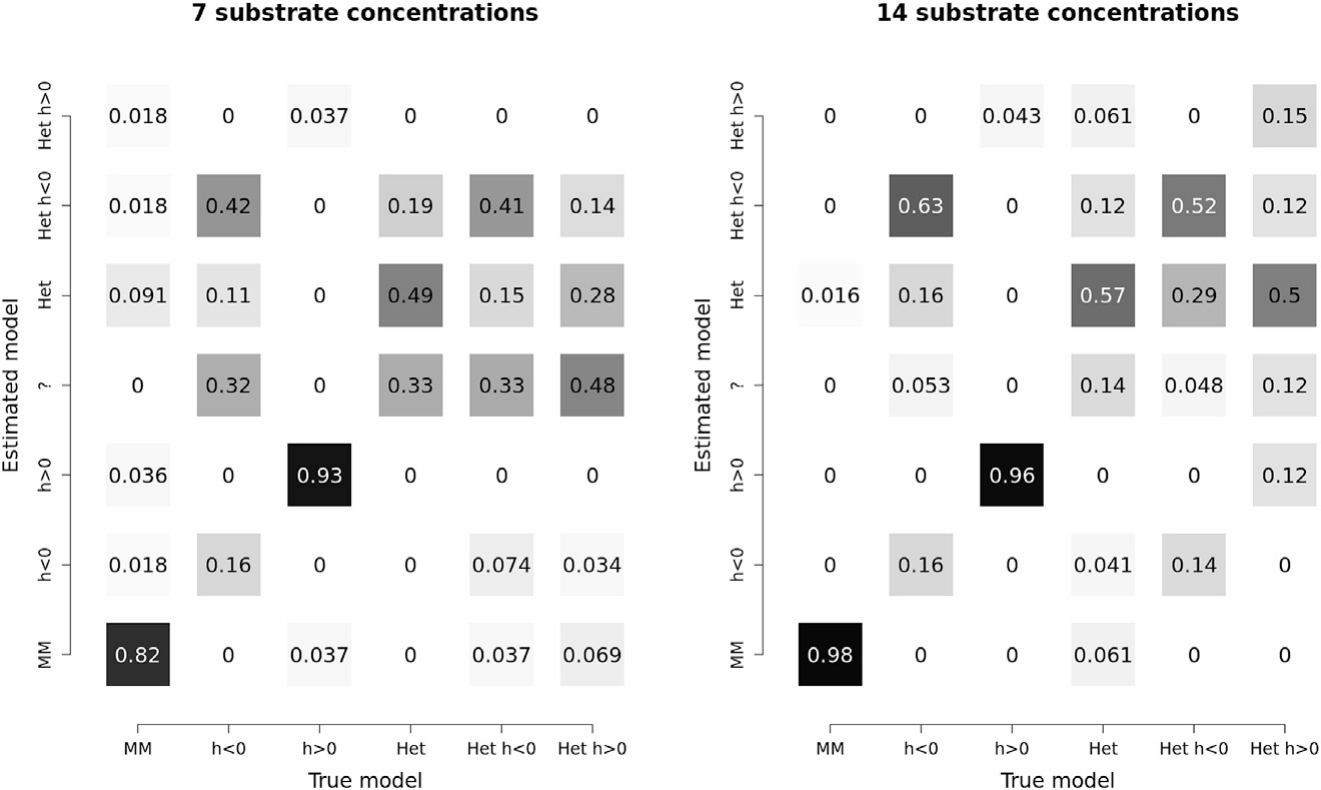
Exploring the interaction between heterogeneous and Hill models Datasets were simulated under six models: homogeneous Michaelis-Menten (MM), homogeneous negative Hill (h>1), homogeneous positive Hill (h>1), heterogeneous Michaelis-Menten (Het), heterogeneous negative Hill (Het h>1), and heterogeneous positive Hill (Het h>1). Bayesian inference was performed on each dataset to estimate the model and its probability. Where any model was identified with greater than 50% posterior support, it is indicated accordingly on the y axis, and where no single model could be identified, it is labeled with a ‘?’. These datasets were simulated with three replicates of seven (left) and fourteen (right) concentrations of substrate, using parameters sampled from the prior. The labeled probabilities are conditional on the true model (i.e., the columns sum to 1). These results suggest that the negative Hill model and heterogeneous model are often non-identifiable and the correct model cannot always be recovered, even on larger datasets, corroborating the findings of Abeliovich 2005.^32^

We also explored the possibility of resolving the newly introduced heterogeneous model from the standard Hill model. To do this, we simulated and performed Bayesian inference on a further 400 datasets, but this time they were simulated under 2×3=6 different models: homogeneous and heterogeneous, crossed with positive, negative, and neutral Hill. Half of these datasets were simulated at 7 substrate concentrations, and the other half with 14 concentrations (3 replicates per concentration). These results suggest that the negative Hill model, which is often interpreted as describing interference between binding sites, is in many cases, indistinguishable from the heterogeneous model, i.e., the two models are non-identifiable (Figure 5). Doubling the number of observations improved the resolution but did not solve the issue. In simple terms, a nonhomogeneous enzyme preparation may give the appearance of an interaction between binding sites, and a system whose binding sites are interacting may give the appearance of an inequality in their catalytic abilities. These two distinct mechanisms are not readily disentangled without further information, or a large volume of high-quality kinetic data.

### Negative control: Homogeneous biological systems

**Figure 6:**
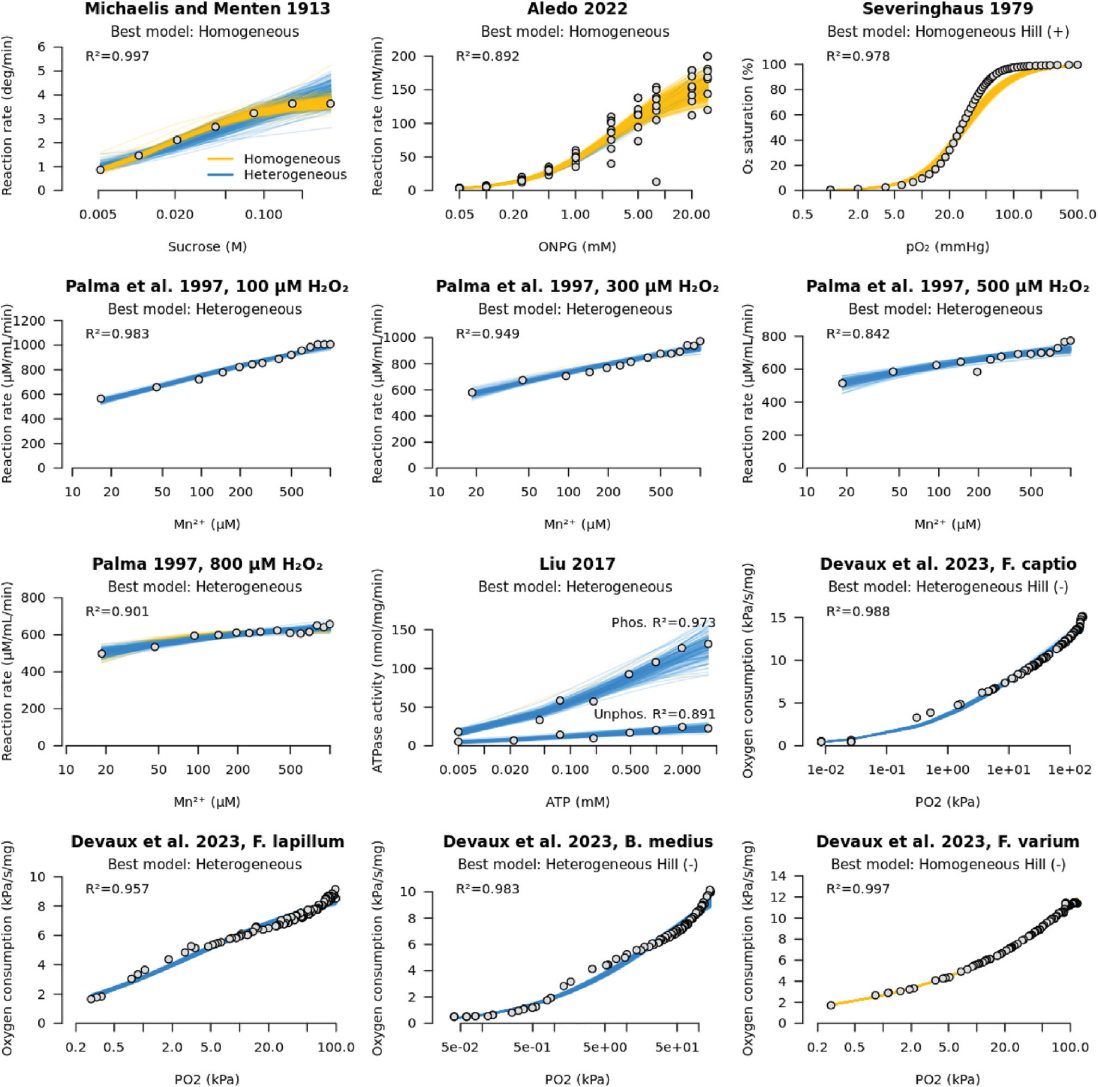
Testing biological datasets for homogeneity and their use of the Hill coefficient Observations are denoted by open circles, and each colored line represents a sampled fit, under the posterior distribution. Yellow lines indicate a homogeneous-model-sampled fit, and blue for heterogeneous. Note that the x axes are on a logarithmic scale. The best model was selected using Bayesian model averaging, and the mean $R^{2}$ is specified for convenience.

We tested our method on three kinetic datasets that were obtained from putatively homogeneous enzyme preparations. In each case, we tested for both homogeneity and use of the Hill coefficient in a joint Bayesian analysis. First, we considered the original experiments carried out by Michaelis and Menten in 1913,^1^ which have since been translated into English.^44^ They measured the hydrolysis of sucrose (into glucose and fructose) by an enzyme then-known as invertase (enzyme commission number EC. 3.2.1.26), across seven sucrose concentrations. Second, we considered the hydrolysis of *o*-nitrophenyl-β-D-galactopyranoside (ONPG) by β-galactosidase (EC. 3.2.1.23). These data are used in the renz package for R,^45^^,^^46^ describing eight experimental replicates, each one carried out by a different group of second-year college students. Third, we considered the human hemoglobin (Hb) dataset from Severinghaus 1979,^47^ which has been described as a “gold standard” for cooperative binding data.^48^ The Hill model is often applied to modeling Hb, as it describes the interaction between its binding sites. These three datasets come with varying standards of experimental precision, as quantified by ϵ (Table 2). The Michaelis-Menten dataset had the lowest experimental error (ϵ=0.033), while unsurprisingly, ONPG, which was aggregated from eight student groups, was comparatively noisy (ϵ=0.35). The 95% credible intervals were reasonably small on all three datasets—usually spanning less than two-fold—making for informative parameter estimates. In all three cases, the homogeneous model was selected (Table 2; Figure 6). Specifically, the ONPG and Hb datasets strongly rejected the heterogeneous model, with $p\left(I_{m}=1\right)<0.05$, while MM was less confident. The Hill model was rejected by MM and ONPG, and selected for Hb, with h=1.72, consistent with positive cooperativity between binding sites. These experiments further corroborate our simulation studies, confirming that our model, and its extra parameter, do not have a tendency to overfit to random noise in the data.

**Table 2:** Testing biological datasets for homogeneity and their use of the Hill coefficient

Dataset		$V_{\max}$	ϵ	$k_{0}$	$\sigma_{K}$	$p\left(I_{m}=1\right)$	$h_{0}$	$p\left(I_{h}=-1\right)$	$p\left(I_{h}=1\right)$
MM^1^		3.92 deg/min (3.56, 4.34)	0.0328 (0.0136, 0.0937)	0.0181 M (0.0144, 0.0504)	–	0.112	–	0.0059	0.0799
ONPG^46^		165 mM/min (140, 192)	0.351 (0.296, 0.405)	2.6 mM (2.03, 3.36)	–	0.0137	–	0	0
Hb^47^		100% (100, 100)	0.189 (0.161, 0.231)	29 mmHg (26.8, 31.3)	–	0	1.72 (1.63, 1.81)	0	1
MnP^49^	100 μM H^2^O^2^	1370 μM/mL/min (1110, 1670)	0.0255 (0.0147, 0.0461)	524 μM (53.6, 3320)	4.43 (2.95, 5.52)	1	–	0	0
	300 μM H^2^O^2^	1120 μM/mL/min (997, 1400)	0.0371 (0.0224, 0.061)	136 μM (10, 828)	4.11 (2.72, 5.75)	0.991	–	0	0
	500 μM H^2^O^2^	787 μM/mL/min (693, 927)	0.0501 (0.0357, 0.0754)	22.2 μM (6.13, 153)	3.63 (1.62, 6.54)	0.991	–	0	0
	800 μM H^2^O^2^	658 μM/mL/min (617, 726)	0.0268 (0.0166, 0.0408)	7.35 μM (2.55, 16.1)	3.02 (0.901, 5.54)	0.922	–	0	0
CFTR^16^	Phos.	190 nmol/mg/min (90.3, 296)	0.169 (0.0841, 0.268)	3.28 mM (0.0228, 31.7)	3.14 (1.87, 4.35)	0.986	–	0.291	0.149
	Unphos.	30.9 nmol/mg/min (20.1, 45.1)	0.214 (0.116, 0.365)	1.67 mM (0.0183, 22.2)	3.52 (2.03, 5.13)	0.986	–	0.297	0.269
Fish^22^	F. captio	21.1 kPa/s/mg (18.1, 25.4)	0.111 (0.0953, 0.128)	102 kPa (22.8, 317)	1.99 (0.856, 2.95)	1	0.525 (0.47, 0.617)	1	0
	F. lapillum	9.54 kPa/s/mg (9.01, 10.2)	0.0538 (0.0469, 0.0626)	11.7 kPa (3.1, 19.4)	2.45 (1.97, 3.03)	0.788	–	0.223	0.294
	B. medius	15.2 kPa/s/mg (12.3, 20.7)	0.102 (0.0898, 0.116)	156 kPa (27.8, 1550)	1.95 (0.92, 3.87)	1	0.552 (0.503, 0.603)	0.56	0
	F. varium	97.3 kPa/s/mg (58.9, 134)	0.0179 (0.0156, 0.0203)	46100 kPa (6510, 155000)	–	0	0.332 (0.324, 0.345)	1	0

Shown are median parameter estimates (and 95% credible intervals), rounded to 3 significant figures. The estimates for $\sigma_{K}$ and $h_{0}$ are omitted when the heterogeneous and Hill models are not being used, respectively.

### Positive control: Heterogeneous biological systems

**Figure 7:**
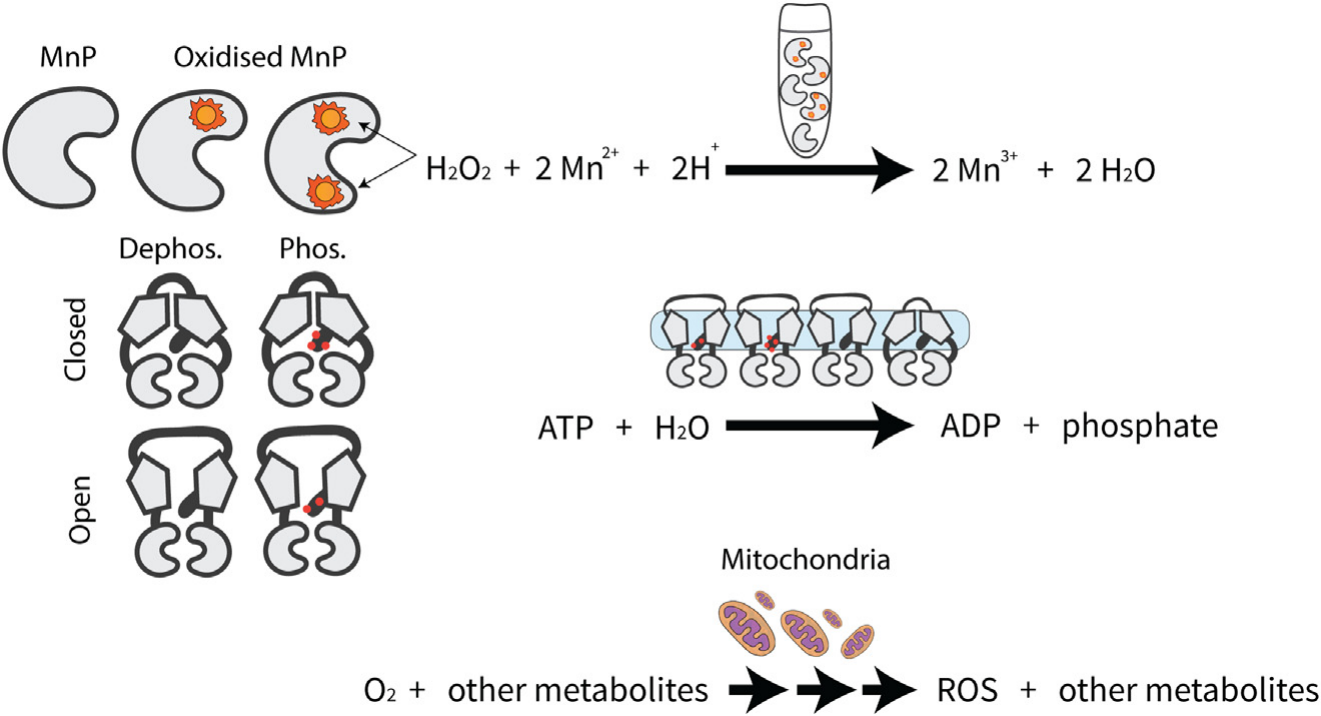
Cartoon summaries of the three putatively heterogeneous systems Top: MnP is oxidized by its substrate H^2^O^2^, resulting in a mixture of enzymes with varying catalytic abilities.^49^ Middle: membrane protein CFTR has heightened ATPase activity when open and phosphorylated, however its varying conformations and varying degrees of phosphorylation may perform this reaction at different effective rates.^16^ Bottom: the fish brain mitochondria, and all their various transport, regulatory, and metabolic processes, consume oxygen and produce reactive oxygen species (ROS) in a non-MM manner.^22^

Finally, we tested our method on three biological systems, each suspected to be nonhomogeneous (Figure 7). These datasets were tested for both homogeneity and use of the Hill coefficient. Where appropriate, the Hill coefficient was heavily constrained *a priori* within $\left(\frac{1}{N},N\right)$ for N binding sites, in accordance with the 1/N rule.^32^ The first two biological experiments were performed under conditions where the enzyme concentration was significantly less than the substrate, and thus the quasi-steady state assumption of the MM model should be satisfied.

First, we considered an enzyme that is degraded by its own substrate. This enzyme is manganese peroxidase (MnP; EC. 1.11.1.13), and it catalyzes the oxidation of Mn^2+^ to Mn^3+^ in the presence of H^2^O^2^. However, H^2^O^2^ is a reactive oxygen species, which decomposes into an even more reactive agent HO^−^ in the presence of metal ions, leading to protein degradation.^19^ Palma et al. 1997 showed that Mn^2+^ exerted a positive effect on enzymatic activity, whereas excess levels of H^2^O^2^ in fact lowered the reaction rate.^49^ We applied our method to initial oxidation rates at varying levels of Mn^2+^, at time zero. These reactions were initiated at four levels of H^2^O^2.^^49^ Our method rejected the assumption of homogeneity in this system. Interestingly, the standard deviation of Michaelis constants declined with increasing doses of H^2^O^2^ (from $\sigma_{K}=4.43$ for 100 μM down to 3.02 for 800 μM), and so did the probability of heterogeneity (from $p\left(I_{m}=1\right)=1$ for 100 μM down to 0.92 for 800 μM). In the last case (800 μM), the heterogeneous model was preferred, but the homogeneous model was still adequate. These results are consistent with reactive products of hydrogen peroxide attacking the protein ensemble, leading to subpopulations of enzymes with severely impaired catalytic activities, while still remaining functional. Greater doses of H^2^O^2^ provided a more uniform degradation effect. As MnP is monomeric with a single active site,^50^ the Hill model was not considered. Variability in Michaelis constant is the best explanation for the non-MM behavior in this system.

Second, we considered a transmembrane ion channel that adopts “open” and “closed” conformations. This channel is the human cystic fibrosis transmembrane conductance regulator (CFTR) protein (EC. 5.6.1.6), and it binds ATP in order to open the channel, enabling the flow of chloride-ions along their electrochemical gradient.^51^^,^^52^ The channel closes upon ATP hydrolysis. The catalytically active (open) state is promoted by phosphorylation of the R domain.^16^ As this is a complex multi-state system, it is reasonable to hypothesize that the active sites may take on a broad distribution of catalytic affinities, and therefore behave heterogeneously. CFTR contains two ATP binding sites,^53^ and therefore its Hill coefficient should be no less than 0.5 and no more than 2 in order to explain cooperative binding. This constraint was reflected in our prior distributions. Indeed, our method favored the heterogeneous model, with h=1, in both datasets by Liu et al. 2017 (phosphorylated and dephosphorylated CFTR). The Hill model was not selected and therefore heterogeneity is a much better explanation for the non-MM behavior displayed by CFTR than cooperative binding. Without invoking a multistate model of CFTR domain rearrangement,^16^ this coarse-grained description of heterogeneity remains the best explanation.

Third, we considered an *in vivo* assay on fish brains. Devaux et al. 2023 evaluated oxygen consumption in the brains of four species of triplefin fish.^22^ The efficiency of the electron transfer chain is likely to vary from mitochondrion-to-mitochondrion and cell-to-cell, and therefore a living tissue like this is likely to be intrinsically nonhomogeneous. In contrast to our previous systems, constraining the Hill coefficient to reflect the number of binding sites is no longer straightforward as this is a living tissue and cooperativity could come in many forms emerging from complex metabolic and regulatory pathways. Moreover, the quasi-steady state assumption of the MM model is often violated in living systems.^3^ Across the four fish brains, our method selected either the heterogeneous model, the Hill model with negative cooperativity, or both. Thus, it is unclear which explanation - heterogeneity, negative cooperativity, or the multi-step reaction chain - is most suitable without further information.

In all three nonhomogeneous systems, our Bayesian approach was unable to reliably estimate the Michaelis means $k_{0}$, whose credible intervals spanned anywhere between 6-fold and over a thousand-fold, thereby rendering the estimates almost meaningless. In contrast, the remaining parameters were estimated more informatively, usually spanning less than three-fold. Our simulation studies did not observe any estimates spanning more than an order of magnitude, suggesting that further work is required to recover Michaelis constants from these systems, for example by developing new models or incorporating more data into the analysis. Estimating the Michaelis constants of heterogeneous enzymes remains an open challenge.

## Discussion

In this article we presented a model that tests for and captures heterogeneity inherent in the Michaelis constants ($K_{m}$) of enzymic systems. The method was validated using simulated data (Figure 3) and then applied to three biological datasets where it greatly outperformed the standard Michaelis-Menten model (Figure 6). This model features an additional parameter $\sigma_{K}$ that describes the standard deviation of Michaelis constants between active sites, which are assumed to be independently drawn from a log-normal distribution. But despite the additional dimensionality, there is no evidence of this model systematically overfitting to random noise in the data. Moreover, the method is quite tolerant to random experimental error. The true model can often be recovered even when the observed reaction velocities span up to one order of magnitude across replicates, but of course less error is always preferred (Figure 3). Corroborating the results of Brown et al.,^7^ we showed that the standard assumption of homogeneity imposed by the Michaelis-Menten model is likely to be sufficient in most cases, and failing only under extreme conditions, such as when the different enzymes’ Michaelis constants span orders of magnitude (Figure 2). Under these conditions, some enzymes must be significantly less proficient than others, but still active, otherwise their catalytic activities would not be detected.

Heterogeneity is often indistinguishable from negative-cooperation under the Hill model^32^; the latter has been widely criticized for lacking a strong theoretical basis.^31^ There exists a twilight zone, in which independently heterogeneous enzymes give the appearance of being non-independently interfering, and vice versa, and the two fundamentally distinct properties give rise to similar hyper-rectangular Michaelis-Menten curves. The two properties are not readily disentangled without further information, such as the number of binding sites per enzyme.^32^ This represents a fundamental limitation in both models, as well as the broader approach of inferring the behavior of molecular processes from two dimensional hyperbolic curves.

We tested six biological systems for their homogeneities and cooperative binding. These systems were (1) sucrose hydrolysis from Michaelis and Menten 1913,^1^ (2) ONPG hydrolysis from second-year college students,^45^ (3) oxygen binding by hemoglobin,^47^ (4) an enzyme (MnP) that is degraded by its own substrate,^49^ (5) an ion channel (CFTR) that adopts multiple conformations,^16^ and (6) oxygen consumption in a living tissue (fish brain).^22^ The first three systems were anticipated to be homogeneous, and our method confirmed this. Whereas, the final three systems were suspected to be heterogeneous. Our results suggested that the MnP and CFTR systems were indeed heterogeneous, and their non-MM-like curves could not be explained by cooperativity between binding sites. The case of the living tissue is more complex and, although the standard MM model was grossly inadequate, we could not unambiguously resolve whether negative cooperation or heterogeneity made a better explanation.

We demonstrated how the assumption of homogeneity can be relaxed in the case of the Michaelis-Menten and Hill models. However, the approach is flexible and may readily be incorporated into other kinetic models, such as the total quasi-steady state approximation.^3^^,^^54^ Although our model of heterogeneity has explanatory power, the approximation of heterogeneity is not based on any first principles (aside from the general observation that many things in nature are approximately normally distributed). In many cases, a specialized model would be preferred, such as a multi-state model for ion channels,^16^ gene regulation,^55^ or transcription elongation.^56^ Such models may also help in yielding informative estimates of the mean Michaelis constant, which could not be reliably recovered in our three biological heterogeneous systems.

Historically, scientists have taken great strides to actively avoid nonhomogeneous enzyme preparations, so as to eliminate as much background noise as possible. However, in some cases heterogeneous preparations may be preferable. For example, one may wish to purify a large sample of computationally modeled enzyme sequences, such as ancestral reconstructions, and screen them simultaneously for catalytic activity. Moreover, capturing variability may be essential for characterizing primordial proteins, which may have existed as populations of “quasi-species”^57^ produced by an ambiguous genetic code.^11^ Our results here have paved the way for these kinds of experiments. Heterogeneity is everywhere in nature and identifying it is only the first challenge. The greater challenge lies in harnessing heterogeneity experimentally and determining when it is biologically meaningful.^21^

### Limitations of the study

This model has limitations. First, heterogeneity cannot be readily distinguished from negatively cooperative binding under the Hill model. Second, our assumption of heterogeneity is captured by a log-normal distribution, however, this approximation of the spread of $K_{m}$ is not based on any first principles. Third, $K_{m}$ is often non-identifiable in heterogeneous systems. Fourth, although the method captures variability in $K_{m}$, it does not capture variability in $V_{\max}$, which is estimated as a weighted average across enzyme species. Despite these assumptions and limitations, we believe this work is a step forward in modeling enzyme kinetics.

## STAR★Methods

### Key resources table

**Table:** 

REAGENT or RESOURCE	SOURCE	IDENTIFIER
**Software and algorithms**		

BEAST v2.7.5	Bouckaert et al. 2019^33^	https://www.beast2.org/
BEAST 2 package: HetMM v0.0.1	This paper	https://github.com/jordandouglas/HetMM
Tracer v1.7.1	Bambaut et al. 2018^39^	https://beast.community/tracer
R v4.3.2	Ihaka and Gentleman 1996^58^	https://www.r-project.org/
R package: HDInterval v0.2.4	Ngumbang et al. 2022	https://CRAN.R-project.org/package=HDInterval
R package: jsonlite v1.8.8	Ooms 2014^59^	https://CRAN.R-project.org/package=jsonlite

### Resource availability

#### Lead contact

Additional information and requests for resources should be directed to, and will be fulfilled by, the lead contact, Dr. Jordan Douglas (jordan.douglas@auckland.ac.nz).

#### Resource availability

No new materials were generated in this study.

#### Data and code availability

• **Dataset**: The fits of our model to the six biological datasets are available as supplementary data.
• **Code**: Our source code is available at https://github.com/jordandouglas/HetMM.
• **Additional information**: Any additional information required is available from the lead contact upon request.

## Supplemental information

Data S1. HetMM fit to MM dataset

Data S2. HetMM fit to ONPG dataset

Data S3. HetMM fit to Hb dataset

Data S4. HetMM fit to MnP 800 µM dataset

Data S5. HetMM fit to MnP 100 µM dataset

Data S6. HetMM fit to MnP 300 µM dataset

Data S7. HetMM fit to MnP 500 µM dataset

Data S8. HetMM fit to CFTR (phosphorylated) dataset

Data S9. HetMM fit to CFTR (dephosphorylated) dataset

Data S10. HetMM fit to Fish F. varium dataset

Data S11. HetMM fit to Fish F. capito dataset

Data S12. HetMM fit to Fish F. lapillum dataset

Data S13. HetMM fit to Fish B. medius dataset
